# Can preoperative modified systemic inflammation score (mSIS) be used to predict malignancy in persistent nondiagnostic thyroid nodules?

**DOI:** 10.3906/sag-2011-177

**Published:** 2021-04-30

**Authors:** Hakan ATAŞ, Birol KORUKLUOĞLU, Bülent ÇOMÇALI, Neşe YAKŞİ, Barış SAYLAM, Mesut TEZ

**Affiliations:** 1 Department of Endocrine Surgery, Ministry of Health Ankara City Hospital, Ankara Turkey; 2 Niğde Community Health Center, Niğde Turkey; 3 Department of General Surgery, Ministry of Health Ankara City Hospital, Ankara Turkey

**Keywords:** Systemic inflammation score, thyroid nodules, malignancy, nondiagnostic, lymphocyte to monocyte ratio

## Abstract

**Background/aim:**

Despite the use of ultrasound guidance, a significant part of thyroid biopsies are nondiagnostic (ND). We aimed to investigate the utility of the preoperative modified systemic inflammation score (mSIS) to predict malignancies in patients with persistent ND thyroid nodules (TNs).

**Materials and methods:**

Records of 924 patients underwent thyroidectomy between September 2016 and May 2019 were retrospectively reviewed. The calculation of mSIS was as follows: mSIS 0 [patients with albumin (ALB) ≥ 4.0 g/dL and lymphocyte to monocyte ratio (LMR) ≥ 3.4], mSIS 1 [ALB < 4.0 g/dL or LMR < 3.4], and mSIS 2 [ALB < 4.0 g/dL and LMR < 3.4].

**Results:**

One hundred and thirty-six patients were included in the study. Of the patients with a median age of 49 (21–81) years, 26 (19.1%) were male, and 110 (80.9%) were female. Besides low lymphocyte count (P = 0.03), and ALB levels (P < 0.01), higher BMI (P = 0.02) were also associated with malignancy. In patients classified as mSIS 2, 1 and 0; malignancy rates were 100%, 25.8%, and 16.1%, respectively. The association between preoperative mSIS and thyroid malignancies was statistically significant (P < 0.01).

**Conclusion:**

We recommend that when patients with persistent ND TNs are assigned to mSIS 2 or 1, surgery should not be delayed due to the risk of malignancy.

## 1. Introduction

Nodular disease of the thyroid gland is common in the community. The incidence of nodules in areas where the disease is endemic can be even more than 50% in high resolution ultrasound (US) examinations [1]. Cytological evaluation is still the most reliable method for suspicious nodules undergoing ultrasound-guided fine needle aspiration (FNA) biopsy to determine the malignancy risk and decision making for surgery. The Bethesda system has been used for this purpose for over ten years and categorizes cytological results according to malignancy risk [2]. However, it may not always be possible to state clearly whether the nodules are benign or malignant as a result of cytological examinations. The Bethesda I category includes inadequate cytological results reported as unsatisfactory or nondiagnostic (ND). The incidence of ND cytology is reported in the literature, ranging from 3% to 36.4% [3]. In cases with ND cytology, the estimated overall risk of malignancy is 5%–10%, while this rate is 9%–32% in surgically treated ones [2]. The first recommendation for patients with ND thyroid nodules (TNs) is to repeat FNA, but in the case of persistent (at least two FNAs) ND cytological results, the clinical features and sonographic findings of the nodules usually determine the clinician’s decision [1,2]. Consequently, the management of persistent ND TNs is often a cause of concern for clinicians, and there is no consensus on whether clinical follow-up or thyroidectomy is the most appropriate approach. Hence, new modalities are needed to distinguish malignant thyroid lesions from benign ones preoperatively, especially for patients with persistent ND nodules.

In the last decade, many studies have been published showing that inflammation plays a crucial role in the development of many types of cancer, including the thyroid [4,5]. Increased thyroid cancer (TC) development in cases of lymphocytic thyroiditis and the effect of immune mechanisms on malignant transformation have been the focus of many articles [4–6]. As such, it is widely believed that measuring of acute phase reactants and certain leukocyte fractions in the blood can be used in the diagnosis of cancer and predicting prognosis [6–8]. Particularly, an increase in serum C-reactive protein (CRP) and decreased levels of serum albumin (ALB) are considered markers of inflammation. These two parameters are the main variables of the modified Glasgow prognostic score used to predict prognosis in colon cancer cases [9]. In addition to serum CRP and ALB, various ratios calculated by circulating leukocyte subgroups such as neutrophil to lymphocyte ratio and lymphocyte to monocyte ratio (LMR) were also evaluated in terms of possible associations with different malignancies [10]. Nevertheless, for patients with TNs, a viable scoring system that could predict thyroid malignancies or a marker evaluated in serum is not currently in use. The systemic inflammation score (both modified and original version) calculated using the preoperative ALB and LMR has been used to evaluate the prognosis in patients with different malignancies and reported to be useful [11,12]. Moreover, a recent study we have conducted provided evidence that modified systemic inflammation score (mSIS) may be useful in predicting malignancy in patients with cytologically indeterminate TNs [13]. Even so, obtained evidence needs to be confirmed in larger case series.

We aimed to investigate the utility of the preoperative mSIS to predict malignancies in patients with persistent ND TNs.

## 2. Material and methods

From September 2016 to May 2019, the records of 924 patients who received thyroidectomy for different indications at the endocrine surgery department of our hospital were retrospectively reviewed. Out of 924 patients, 136 patients with ≥2 ND cytological results were included in the study. ND TNs with subsequent different cytologies were excluded from the study. 

All FNAs were performed with a 25–27 gauge needle and 20 mL syringe under US guidance by experienced endocrinologists. Consecutive FNA procedures were performed with an average interval of 3 months. The specimens were not evaluated on-site for sufficiency. FNA samples were evaluated by cytopathologists according to the Bethesda system, and specimens showing less than six groups of cells that consisted of less than ten cells each group were defined as ND. Patients with nodules larger than 4 cm in diameter, those who had previously received radiation to the neck region, and those with histopathologically proven thyroiditis were excluded from the study. Other exclusion criteria were as follows; active infection in the last three months, steroid use, systemic inflammatory diseases such as Crohn’s or ulcerative colitis, liver failure, other cancer, renal failure, and acute myocardial infarction in the last six months. The patients included in the study were divided into two groups named malignant and benign, considering the final pathology results. Demographic data such as age, sex, body mass index (BMI), which were thought to be associated with the development of malignancy, and preoperative thyroid-stimulating hormone (TSH), white blood cell counts, ALB and thyroid volume (TV) data were recorded. The formulas used for variables that require special calculations were as follows; BMI = weight (kg) / height squared (m2), TV (elliptical shape volume) = Length × width × height × 0.479 (for each lobe) and LMR = total count of lymphocytes / total count of monocytes. Blood samples were collected following a fast for at least 8 h and studied over a 30-min period. The mSIS was calculated for patients following the determination of malignancy-related variables. The formulation for mSIS was as below [12]. 

ALB ≥ 4.0 g/dL and LMR ≥ 3.4 →mSIS 0

ALB < 4.0 g/dL or LMR < 3.4 →mSIS 1

ALB < 4.0 g/dL and LMR <3.4 →mSIS 2 

Then, patients were divided into three groups according to the number of mSIS, and the relationship between changing score and malignancy was statistically evaluated.

### 2.1. Statistical analysis

Statistical analyses were performed using IBM Statistical Package for the Social Sciences version 21 (IBM SPSS Corp.; Armonk, NY, USA). The Kolmogorov–Smirnov and Levene’s tests were used to assess the normality and the homogeneity of data distribution, respectively. The mean ± standard deviation (SD) values were used to describe for normally distributed continuous variables, and median (min-max) were used for nonnormally distributed ones. Differences in continuous variables were analyzed using the Student’s t-test, Mann–Whitney U test, or Kruskal–Wallis test, χ2 tests or Fisher’s exact test were used to analyze the categorical variables. The binary logistic regression method was used to evaluate the factors affecting malignancy. The variables that significantly affect the pathology results in univariate analyses and variables that make a significant difference in the literature were included in the model. Variables showing multicollinearity were not included in the model. Statistical significance was calculated at the 95% confidence interval, and a P-value <0.05 were considered significant for the presented results.

## 3. Results

Fine needle aspiration cytology (FNAC) results were persistent ND in 147 (15.9%) of 924 patients undergoing thyroidectomy. However, eleven patients were excluded from the study due to a history of malignancy, renal failure, and the presence of inflammatory diseases. One hundred and thirty-six patients were included in the study and the median age was 49 (21–81) years. Of these, 26 (19.1%) were male and 110 (80.9%) were female. The median value for the patients’ BMI was 28.8 (19.7–45.5) kg/m2. The mean size of the ND nodules’ was 15.30 ± 0.95 mm (9–39 mm). While thyroidectomy was performed in 115 cases totally, only 21 cases underwent lobectomy. As a result of histopathological evaluation of surgical specimens, 39 patients (28.6%) were diagnosed as malignant. Micropapillary carcinoma was identified in 10 patients (25.6%), papillary carcinomain 27 patients (69.2%) and follicular carcinoma in 2 patients (5.2%). Demographic and laboratory data of benign and malignant groups are presented in Table 1.

**Table 1:** Univariate analyses for benign and malignant groups.

Characteristics	Benign (n = 97)n (%)*	Malignant (n = 39)n (%)*	P-value
Age (years)	49 (21–71)	48.5 (26–81)	0.58§
SexFemaleMale	81 (83.5)16 (16.5)	29 (74.4)10 (25.6)	0.22‡
BMI (kg/m2)	28.3 (19.9–40.6)	31.4 (19.7–45.5)	0.02§
Thyroid volume, mL	43.1 ± 35.9	46.2 ± 39.7	0.91†
TSH, mIU/mL	1.1 ± 0.8	1.3 ± 1.2	0.57†
Lymphocyte (/µL)	2.2 (1.2–5.9)	1.9 (0.8–3.4)	0.03§
Monocyte (/µL)	0.4 ± 0.1	0.5 ± 0.1	0.09†
LMR	5.6 ± 4.9	3.8 ± 1.4	0.05†
ALB, g/dL	4.3 (3.4–5.1)	3.9 (3.7–4.7)	<0.01§

*Mean ± standard deviation for a normally distributed continuous variable, median (min-max) for nonnormally distributed continuous variables were demonstrated. † Student’s t-test, ‡χ2 tests, §Mann–Whitney U test.BMI: body mass index, TSH: thyroid-stimulating hormone, LMR: lymphocyte to monocyte ratio, ALB: albumin.

In univariate (UV) analyses, there was no difference between benign and malignant groups in terms of age and sex. However, it was striking that in the malignant group, BMI was significantly higher (P = 0.02), and ALB values and lymphocyte count were significantly lower (P < 0.01 and P = 0.03, respectively). According to the results of UV analysis, a model was created for multivariate analysis. It was found that LMR statistically affected the pathology results if the effects of BMI and ALB on pathology results were controlled (OR: 0.66, 95% CI: 0.45–0.95) (Table 2).

**Table 2:** Multivariate analyses of the parameters affecting pathology results.

	Odds ratio	95% CI	P-value*
BMI (kg/m2)	1.12	1.03–1.23	0.01
ALB (g/dL)	0.05	0.01–0.26	< 0.01
LMR	0.66	0.45–0.95	0.02

*Binary logistic regression. CI: confidence interval.

Of 136 patients with persistent ND cytology, 87 (63.9%) were assigned as mSIS 0, 35 (25.8%) as mSIS 1 and 14 (10.3%) as mSIS 2. In patients classified as mSIS 2, 1 and 0; malignancy rates were 100%, 25.8%, and 16.1%, respectively. The malignancy rate was significantly higher in mSIS 2 group compared to mSIS 1 and 0 group (P < 0.01); however, tumor size did not differ between the mSIS groups (P = 0.89) (Table 3, Figure). The parameters for the validity of mSIS in predicting malignancy for different risk groups are presented in Table 4.

**Table 3:** Comparison of final pathology results according to the modified systemic inflammation score (mSIS).

	mSIS 0n (%)*	mSIS 1n (%)*	mSIS 2n (%)*	P-value
Final pathology results				
Benign	73 (83.9)	24 (68.6)	0 (0.0)	<0.01†
Malignant	14 (16.1)	11 (31.4)	14 (100.0)	
Malignant tumor diameter (mm)	11.5 (9–23)	14.0 (9–28)	13.5 (10–39)	0.89‡
Malignant tumor classification (n = 39)				0.90†
Microcancer	4 (28.6)	3 (27.3)	3 (21.4)	
Macrocancer	10 (71.4)	8 (72.7)	11 (78.6)	

* Median (min-max) for nonnormally distributed continuous variables were demonstrated. † χ2 tests,‡Kruskal–Wallis test.

**Figure:**
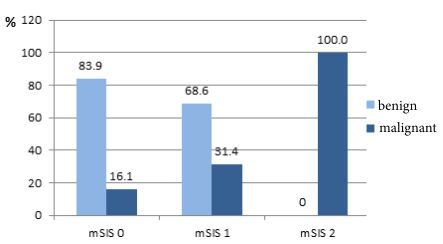
Distribution of pathology results by mSIS groups.

**Table 4:** The parameters for the validity of mSIS in predicting malignancy for different risk groups.

High-risk default group for malignancy	Sensitivity(%)	Specificity(%)	PPV(%)	NPV(%)	Accuracy rate (%)
mSIS 1 or 2	64	75	51	84	72
mSIS 2	35	100	100	79	82

PPV: positive predictive value, NPV: negative predictive value.

## 4. Discussion

The management of persistent ND TNs is often controversial. International guidelines recommend repeating FNAC with ultrasound guidance or excision with diagnostic surgery, which is generally in favor of lobectomy [2]. However, since the majority of ND nodules are not removed, it is difficult to calculate and interpret the risk of malignancy. This is another reason for the with controversy over the extent of the surgery to be performed. Therefore, in addition to cytological evaluation and US features, it is obvious that a new diagnostic scoring method is needed to predict the malignancy risk of ND nodules and to decide the most appropriate one from surgical and follow-up options.

Given the relationship between inflammation and carcinogenesis, the idea that blood markers of inflammation can also be used as a biomarker for cancer diagnosis and post-treatment follow-up is not far off [4,5,8]. However, most of the studies to date show that inflammation markers are used to determine the prognosis of TC, and the number of studies focusing on the diagnosis of the disease is very few [6,7]. In the current study, the malignancy rate of patients with ND nodules who had only histological follow-ups and surgically treated was 28.6%. The rate we found was in accordance with the rate of malignancy reported in the literature for resected ND nodules [2]. We used mSIS in our study to predict the presence of cancer. We demonstrated that the preoperative score and malignancy rates correlated in patients with ND nodules. We even found that all patients with mSIS 2 had malignancy. The feature of mSIS is that it evaluates three important components (ALB, lymphocyte and monocyte) that are predicted to be related to inflammation and carcinogenesis. When inflammation becomes chronic, ALB synthesis is reduced in the liver, indicating malnutrition and cachexia in cancer cases [14,15]. Also, lymphocytes are very important in cancer immunity, and their decrease in number is associated with a poor prognosis. Another important issue is the decrease in the number of monocytes that differentiate into macrophages in circulation and accelerate cancer development by contributing to vascularization, growth and metastasis of the tumor [4,16]. As a result, mSIS calculated using ALB and LMR can accurately reflect the immune response that points out carcinogenesis.

In a recent article, researchers demonstrated the relationship between ALB level below 40 g/L and thyroid malignancies, however, this relationship was not observed for white blood cells, haptoglobin and CRP [17]. A study conducted to distinguish TCs from benign nodules provided evidence that both increased platelet distribution width (PDW) and low ALB level may be associated with TCs [7]. Another study revealed that decreased LMR might be associated with increased recurrence in differentiated thyroid malignancies [8]. Several authors have suggested that low ALB levels may be associated with thyroid malignancies. However, different and discrepant results on other inflammation markers suggest that there is still no consensus on this issue.

In literature, many studies aiming to predict preoperative malignancy in patients with ND TNs are generally concentrated on Thyroid imaging reporting and data system (TI-RADS) [3,18]. In their study of 246 patients with persistent ND TNs, Başer et al. could not reveal any suspicious ultrasonographic features for predicting malignancy or did not point out any difference between benign and malignant nodules in terms of TI-RADS scores [3]. However, Yoon et al. concluded that the malignancy risk stratification with TIRADS for ND TNs is more effective than the Bethesda system. Their suggestion was to follow-up with US-guided FNAC for TNs with TIRADS category 4b, 4c, or 5. Nevertheless, they could not provide an acceptable new recommendation for estimating malignancy risk for patients with persistent ND TNs [18].

Another point revealed by the present study was that one of the factors associated with malignancy in patients with persistent ND TNs was high BMI. Another point revealed by this study is that a high BMI of patients with persistent ND nodules is another factor associated with thyroid malignancies. Recently, some authors have published a quite large series showing the positive association between higher BMI and TC risk [19]. However, there is no sufficient data in the literature that higher BMI increases malignancy in patients with persistent ND TNs. Therefore, it is obvious that new studies will clarify whether BMI is a risk factor associated with malignant transformation of ND TNs.

The limitations of this study can be expressed as follows. First, the study design is retrospective, and the sample size is small since only patients with persistent ND nodules are included. Therefore, this situation may have reduced the statistical power of some subgroup analyzes. Another important point is that it is unavailable due to the retrospective nature of the study and is likely to affect mSIS data such as diet and drug use.

In our study, we evaluated the patients who underwent thyroid surgery due to both suspicious ultrasound findings (such as solidity, hypoechogenicity, irregular margins, taller-than-wide shape, and microcalcifications) and persistent ND cytology. Therefore, the results we obtained should not be generalized for all patients with ND cytology. However, to our knowledge, our study is the first report in the literature that provides evidence between mSIS and the malignancy risk of persistent ND TNs. The patients included in the study belong to the most controversial and challenging category of the Bethesda classification. In this respect, the results obtained from our study will contribute significantly to clinicians and endocrine surgeons in decision making.

In conclusion, this study indicates that high mSIS in patients with persistent ND cytology may be associated with thyroid malignancies. Although promising in terms of specificity, mSIS may not be considered as an ideal scoring to be used alone to predict the presence of malignancy as its sensitivity is not high enough. Nevertheless, due to its low cost and easy applicability, mSIS can be used as an adjunctive method to determine the malignancy riskof persistent ND nodules. Our results strongly support that, because of the increased risk of malignancy, surgery should not be delayed if patients with persistent ND TNs are assigned to mSIS 2 or 1, particularly in endemic goiter regions. Even so, further studies are needed to confirm these preliminary results.

## Informed consent

This study was approved by the Institutional Review Board of Ankara City Hospital Ethics Committee (Number: E-1-20-697;May 28, 2020). Informed consent was taken from all patients during the registration. All procedures in this study involving human participants were performed in accordance with the 1964 Helsinki Declaration and its later amendments.
